# Development of a SYBR Green I real-time PCR for the detection of the orf virus

**DOI:** 10.1186/s13568-016-0322-9

**Published:** 2017-01-07

**Authors:** Yong Wang, Kankan Yang, Caixia Bai, Dongdong Yin, Gang Li, Kezong Qi, Guijun Wang, Yongdong Li

**Affiliations:** 1 College of Animal Science and Technology, Anhui Agricultural University, Hefei, 230036 People’s Republic of China; 2 Municipal Key Laboratory of Virology, Ningbo Municipal Center for Disease Control and Prevention, Ningbo, 315010 People’s Republic of China; 3 State Key Laboratory of Animal Nutrition, Beijing Institute of Animal Science and Veterinary Medicine, Chinese Academy of Agricultural Sciences, Beijing, 100193 People’s Republic of China

**Keywords:** Detection, Orf virus, Real-time PCR, SYBR Green I

## Abstract

Orf is a non-systemic, ubiquitous disease of sheep and goats caused by the orf virus (ORFV). ORFV occasionally causes cutaneous lesions in humans in contact with infected animals. In the present study, a real-time PCR method was established for detection of ORFV using the fluorescent chimeric dye SYBR Green I. Specific primers were designed to target a highly conserved region of the ORFV B2L gene. This method was able to detect a minimum of 20 copies of ORFV genomic DNA. The results showed no cross-reactions with other common DNA viruses. The time required for the test was approximately 1.5 h. Clinical test samples showed that this method was faster and had a higher sensitivity than traditional PCR. In conclusion, this novel, real-time PCR-based assay provides a rapid, sensitive, and specific method for ORFV detection. This test provides improved technical support for studies regarding the clinical diagnosis and epidemiology of ORFV.

## Introduction

Orf, also known as contagious ecthyma or scabby mouth, is a non-systemic, ubiquitous disease of sheep and goats caused by the orf virus (ORFV). It occasionally causes cutaneous lesions in humans who contact infected animals. ORFV is a member of the *Parapoxvirus* genus and the *Poxviridae* family (Friebe et al. 2011). The disease is characterized by pustular lesions around the mouth and nostrils. Although the mortality rate is low in adult animals, the morbidity rate is high. In immunosuppressed animals and in lambs, orf may be fatal (Gumbrell and McGregor 1997), with a reported mortality rate in lambs and kids of up to 10 and 93%, respectively (Hosamani et al. 2007).

Reports of severe orf outbreaks have gradually increased in recent years (Abrahao et al. 2009; Oem et al. 2009; Zhao et al. 2010). Therefore, it is critical that ORFV infection be quickly and accurately identified. To differentiate ORFV infection from infection by other pathogens (including goatpox virus and sheeppox virus), laboratory tests such as viral isolation, electron microscopy, counter-immuno electrophoresis, serum neutralization tests, ELISA, and PCR have been used for diagnosis. However, these traditional methods often are associated with drawbacks, such as low detection sensitivity or time-consuming protocols. Therefore, the development of a rapid, specific, and effective method for detection of ORFV in high-throughput clinical samples would offer advantages over current methods.

In the present study, we established a real-time PCR detection method based on the chimeric fluorescent dye SYBR Green I. It can be used for rapid, sensitive, and specific detection of ORFV and includes a simple protocol with high-throughput capability. This novel method may be suitable for epidemiological research and clinical diagnosis.

## Materials and methods

### Viruses and cultures

The ORFV was isolated from scab specimens collected from skin lesions of a 1-month-old goat affected with ORFV in November 2015 in Anhui province of China. The scabs were collected and suspended in PBS (pH 7.2). After culture on primary ovine fetal turbinate (OFTu) cells for ten passages, the virus was named ORFV AH-F10 and maintained in liquid nitrogen for preservation.

The procedures for obtaining and culturing the primary OFTu cells are shown below. Briefly, after the pregnant goat was anesthetized, the abdomen and uterus were surgically opened. The goat embryos were removed in a sterile manner. The turbinate parts of the embryos were washed in the PBS (pH 7.2) three times. Then, the turbinate parts were cut into small pieces in serum-free Dulbecco’s modified Eagle medium (DMEM; Thermo Fisher Scientific, Waltham, MA, USA). The turbinate parts were digested with trypsin at a concentration of 12.5 g/L at 37 °C for 10 min in a water bath. Then, the digested products were centrifuged at 1000 r/min for 15 min. The cells were collected and added into complete serum containing 10% fetal bovine. The cell suspension was counted and diluted into 5 × 10^5^ cells/μL. OFTu cells were cultured in 6-well plates (400 µL per well) and incubated for 1 h at 37 °C under 5% CO_2_. ORFV was added to the OFTu cells, and the cells were gently shaken for 10 min three times before the liquid was removed. Next, 2 mL of DMEM containing 2% fetal bovine serum (FBS) was added to each well. After 72 h, OFTu cells exhibited typical cytopathic effects (CPEs). The cells were then freeze-thawed three times. After centrifugation at 2000*g* for 5 min, the supernatants were collected and used for DNA extraction. Goatpox virus, sheeppox virus, and swine pseudorabies virus were propagated in baby hamster kidney cells (BHK-21) in DMEM containing 10% heat-inactivated FBS and 1% antibiotics (100 U/mL penicillin and 100 mg/mL streptomycin).

### Primer design

Primer sequences are shown in Table 1. Specific primers (F2, R2) were designed to target a conserved region of the ORFV B2L gene and were used to obtain the target sequence (180 bp) through real-time PCR. Primers F1 and R1 were used for conventional PCR amplification of a 514-bp region of the B2L gene of the ORFV AY424972 strain. The above primers and nucleic acid sequences were analyzed by BLAST searches against GenBank (http://www.ncbi.nlm.nih.gov/).

**Table 1:** Primer sequences and probes designed in the study

Primer	Sequence (5′ → 3′)	Genomic position	Product length (bp)
F1 R1	CGGAATTCAGTCCGCGAAGAAGTTTTTG CCCTCGAGGCGAGTCCGAGAAGAATACG	125 622	514
F2 R2	GGGCTCTACTCCACCAACAA CGAGTCCGAGAAGAATACGC	442 621	180

### Preparation of standard DNA

Orf virus DNA was extracted from OFTu cells infected with ORFV using the DNeasy Tissue Kit (Qiagen, Hilden, Germany) according to the manufacturer’s instructions. DNA was eluted with 100 μL of DNase−, RNase−, and protease-free water (Life Technologies, Gent, Belgium). DNA integrity was verified on a 2% agarose gel, and DNA was stored at −80 °C until use. The B2L gene was amplified using conventional PCR (primers F1 and R1) with Premix Taq (Takara Bio Inc., Shiga, Japan). The annealing temperature, extension time, and primer concentration of the traditional PCR were optimized. The PCR program included 30 cycles of 30 s at 95 °C for denaturation, 30 s at 55 °C for annealing, and 1 min at 72 °C for extension, followed by a final extension at 72 °C for 10 min. PCR products were detected by electrophoresis on a 2% (w/v) agarose gel stained with ethidium bromide. PCR products were purified using a gel extraction kit (Omega Bio-tek, Norcross, GA, USA).

Purified PCR products were inserted into a pBluescript II SK(6) plasmid (Stratagene, Santa Clara, CA) after treatment with the restriction enzymes *Eco*RI and *Xho*I (New England Biolabs, Ipswich, MA, USA). The recombinant plasmid was verified, transformed into DH5α competent cells, extracted with Mini Kit I (Omega Bio-tek), and sequenced. The concentration of the extracted plasmid was determined using a NanoDrop 2000 system (Thermo Fisher Scientific) and used to calculate the number of copies of the plasmid (copies/μL). Aliquots of DNA were prepared in ten-fold serial dilutions from 1 × 10^8^ to 1 × 10^1^ copies/μL and stored at −20 °C until use.

### Establishing a standard curve for real-time PCR

Real**-**time PCR was performed using SYBR Premix Ex Taq II (Takara). Each reaction contained 20 μL of the following ingredients: 10 μL of 2 × SYBR qPCR Mix, 0.8 μL each of forward and reverse primers (10 μM F2 and R2), 2 μL of template DNA, and 6.4 μL of ddH_2_O. Real-time PCR was conducted using a real-time PCR detection system (Bio-Rad, Hercules, CA, USA) with the following program: 95 °C for 30 s, followed by 40 cycles of 95 °C for 5 s and 60 °C for 1 min. PCR for each template and the negative control was repeated three times.

### Specificity assay

The specificity of the real-time PCR assay was evaluated using several DNA viruses including goatpox virus (Accession number: AY077836.1), sheeppox virus (Accession number: AY077833.1), and swine pseudorabies virus (HB-98 vaccine Strain). The specificity assay was performed using the conditions and systems described above and repeated three times with each template and the negative control. At the same time, traditional PCR was conducted for comparison with real-time PCR.

### Sensitivity assay

The standard DNA stock was serially diluted ten times with sterile water to concentrations of 1 × 10^8^–1 × 10^1^ copies/μL to determine the lower detection limit, and conventional PCR (1 × 10^9^–1 × 10^1^ copies/mL) was performed using primers F1 and R1. Each reaction tube contained 2 μL of DNA templates and was tested using real-time PCR and conventional PCR. Standard DNA and the negative control samples were amplified three times each. The concentrations of standard DNA templates for conventional PCR and real-time PCR were the same. PCR products were analyzed using agarose gel electrophoresis.

### Reproducibility assay

To determine the reproducibility of real-time PCR, standard DNA was diluted to 1 × 10^7^, 1 × 10^5^, 1 × 10^3^, and 1 × 10^1^ copies/μL. Each reaction tube contained 2 μL of template. Intra- and inter-assay reproducibility was assessed using the conditions and systems described above. Intra-assay and inter-assay variation was assessed in three replicates for each concentration in a single round of real-time PCR. Experiments were repeated three times on different days. The mean, SD, and coefficient of variation were calculated separately using threshold cycle (*C*_*t*_) values to determine the intra-assay and inter-assay reproducibility.

### Detection in samples from naturally infected goats

To confirm the applicability of the real-time PCR in clinical diagnostics, DNA was prepared using the Qiagen DNA Tissue Kit (Qiagen) according to the manufacturer’s instructions. Nose and mouth secretion samples were derived from 65 goats from two goat farms whose clinical symptoms suggested infection with ORFV. There were 30 male goats and 35 female goats. The goats were at the age of 1–6 months, and had developed proliferative lesions around the mouth and lips. The lesions were nodular, with a size of approximately 4–10 mm. The tissue samples were scraped and were then divided into two sections. One was preserved at −70 °C and the other was used for DNA extraction. The samples were dissolved in PBS (pH 7.4) according to the rate w/v = 1 mg/mL and then suspended in PBS (pH 7.4) with penicillin and streptomycin. Then, extracted DNA was used for real-time PCR and conventional PCR detection. Positive samples were used to infect OFTu cells for further observation of CPEs.

## Results

### Establishment of a standard curve for real-time PCR

**Fig. 1:**
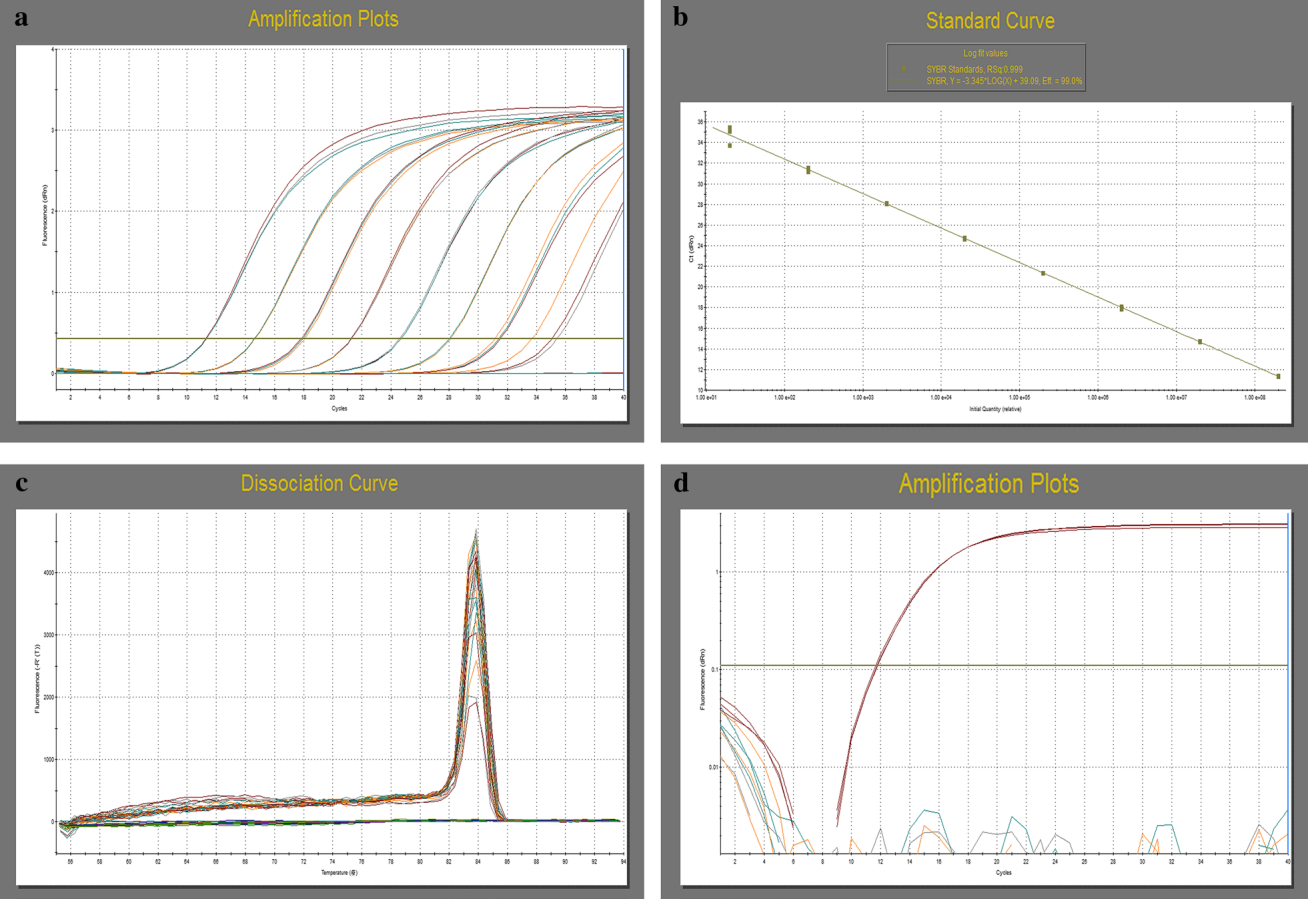
Results of SYBR Green I real-time PCR for standard ORFV DNA. **a** Amplification curve. Ten-fold dilutions of standard DNA ranged from 1 × 10^8^ to 1 × 10^1^ copies/µL. **b** *Standard curve*. Dilutions ranged from 2 × 10^8^ to 2 × 10^1^ copies per reaction. *Standard curve*: y = − 3.345 × log (x) + 39.09; correlation coefficient: R^2^ = 0.999; reaction efficiency: Eff = 99.0%. **c** *Melting curve*. Dilutions ranged from 2 × 10^8^ to 2 × 10^1^ copies per reaction. Tm = 83.79 °C. **d** Specificity of SYBR Green I real-time PCR. *Sample 1* orf virus, *2* goatpox virus, *3* sheeppox virus, *4* swine pseudorabies virus, *5* no template (negative control)

The full-length B2L gene was inserted into the pBluescript II SK(6) vector after treatment with the restriction enzymes *Eco*RI and *Xho*I. The recombinant plasmid was then successfully verified using double restriction digest and PCR. The length of the pBluescript II SK(6)-B2L gene was 3435 bp, and the concentration of the standard DNA was determined to be 218 ng/μL. Copy number was calculated as 5.79 × 10^10^ copies/μL [6.02 × 10^23^ × 218 ng/μL × 10^−9^/(3435 bp × 660 Da/bp)]. The standard DNA was diluted to 1 × 10^8^–1 × 10^0^ copies/μL with ddH_2_O. Each real-time PCR reaction used 2 μL of template, and each concentration was amplified three times to detect ORFV (Fig. 1a). The standard curve was determined to be y = −3.345 × log(x) + 39.09, and the efficiency was calculated to be 99.0% (Fig. 1b). Melting curve analysis revealed a single peak for the template at 2 × 10^8^–2 × 10^0^ copies (Fig. 1c).

### Specificity of real-time PCR

The specificity of real-time PCR was examined using goatpox virus, sheeppox virus, and swine pseudorabies virus. Although ORFV DNA was amplified by real-time PCR, no other pathogen DNA tested was amplified. This demonstrates that the established method for real-time PCR exhibited no cross-reactions with other common pathogens but rather exhibited high specificity (Fig. 1d).

### Sensitivity of real-time PCR

**Fig. 2:**
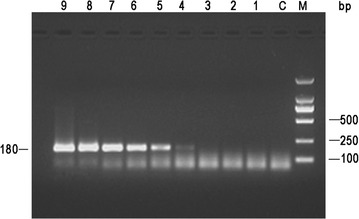
Sensitivity of conventional PCR using ORFV DNA as templates. Ten-fold dilutions of standard DNA ranged from 2 × 10^9^ to 2 × 10^1^ copies/µL.* C* negative control; *lanes 1–9* standard DNA (2 × 10^1^–2 × 10^9^ copies per reaction);* M* DL 2000 marker

Conventional PCR and real-time PCR assays were conducted to test the sensitivity using the standard DNA as a template. The real-time PCR was able to detect a minimum of 20 copies of DNA. Moreover, at the peak of the single melting curve, there was no non-specific amplification (Fig. 1c). The lower detection limit of the conventional PCR was 2 × 10^4^ copies of DNA template (Fig. 2). These results indicate that the sensitivity of the real-time PCR method is approximately 1000 times higher than that of the conventional PCR.

### Intra- and inter-assay reproducibility of real-time PCR

Standard DNA was diluted to 1 × 10^7^, 1 × 10^5^, 1 × 10^3^, and 1 × 10^1^ copies/μL to assess the intra- and inter-assay reproducibility of the real-time PCR. Intra- and inter-assay Ct values were used to calculate the mean, SD, and CV. The inter- and intra-CV values ranged from 0.16 to 0.39% and 0.11 to 0.21%, respectively, indicating that the assay was highly reproducible (Table 2).

**Table 2:** Intra- and inter-reproducibility assay

Category	DNA standard (copies/µL)	Mean (Ct)	SD	CV (%)
Intra-assay	1 × 10^7^	15.09	0.017	0.11
	1 × 10^5^	21.65	0.025	0.12
	1 × 10^3^	28.55	0.030	0.11
	1 × 10^1^	35.17	0.075	0.21
Inter-assay	1 × 10^7^	15.15	0.041	0.27
	1 × 10^5^	21.76	0.035	0.16
	1 × 10^3^	28.44	0.048	0.17
	1 × 10^1^	35.15	0.136	0.39

### Detection of ORFV in naturally infected goats

In total, 65 clinical samples from goats suspected of having ORFV infection were collected from different geographical locations of Anhui province from February to October 2016. The DNA extracted from the scab material was used as a template for evaluation of the established assay. Conventional PCR and real-time PCR were performed simultaneously to compare the sensitivity of the two detection methods. The total positive detection rate by conventional PCR was 66.15% (43/65), whereas that of the real-time PCR was 86.15% (56/65) (Table 3). The highest concentration of ORFV detected in an individual sample was 10^7^ copies/g. Among the clinical samples, all samples that tested positive by conventional PCR were positive by SYBR Green real-time PCR (data not shown). The sequences of amplicons from samples that were positive using real-time PCR but negative in conventional PCR subjected to a BLAST search in the NCBI database. The sequences were consistent with the B2L gene sequence of ORFV, confirming the higher sensitivity of the real-time PCR over the conventional PCR method.

**Table 3:** Detection of orf virus in clinical samples by conventional PCR and real-time PCR

Regions of Anhui province	Number of clinical samples	Conventional PCR positive (%)	Real-time PCR positive (%)
Feidong	15	60.00 (9/15)	86.67 (13/15)
Bozhou	16	62.50 (10/16)	81.25 (13/16)
Jinzhai	16	68.75 (11/16)	87.50 (14/16)
Shouxian	18	72.22 (13/18)	88.89 (16/18)
Total positivity	65	66.15 (43/65)	86.15 (56/65)

## Discussion

In this study, we developed a sensitive and specific SYBR Green I real-time PCR assay to quantitatively detect ORFV. Viral detection can utilize several different methods. Viral isolation is considered to be the gold standard for detection of pathogen infection. Endpoint dilution assays and plaque assays have also been used to quantify viruses. Compared with these methods, the SYBR Green I real-time PCR assay developed in this study offers several advantages, including more rapid results, reduced labor, and high-throughput capability. Moreover, viral isolation, endpoint dilution assays, and plaque assays are only able to identify infectious viral particles. In the case of field isolates that have not been adapted for growth in vitro, viral quantification via such methods is difficult.

A conventional PCR assay based on amplification of the ORFV B2L gene was developed to detect many recognized *Parapoxvirus* species (Gallina et al. 2006; Sullivan et al. 1994). When combined with DNA sequencing, this method can be used to distinguish among the different *Parapoxvirus* species (Inoshima et al. 2000). However, conventional PCR methods are not quantitative and can sometimes include non-specific products of the same size. To avoid these issues, a real-time PCR assay based on SYBR Green I was developed for the detection and quantification of ORFV. Many studies have demonstrated that real-time PCR is accurate and effective in quantifying viral DNA (Espy et al. 2000; Liu et al. 2013; Lo and Chao 2004; Mohamed et al. 2013; Niesters 2001). Even in the case of moderate DNA quality, such as DNA extracted from paraffin- or formalin-fixed tissues, real-time PCR can be effectively used for viral detection (Lu et al. 2016; Norlelawati et al. 2016). Compared with conventional PCR, a real-time PCR-based assay eliminates the requirement for gel electrophoresis of the reaction product. Reaction results can instead be observed in real time and clearly interpreted. Another advantage to the real-time PCR method is that the entire process requires only approximately 1.5 h, making it particularly suitable for high-throughput detection of clinical and laboratory pathogens.

Generally, conventional PCR assays often involve the amplification of long DNA fragments, reducing their sensitivity. To improve the sensitivity, we designed our assay to produce a short amplicon of 514 bp, enabling sensitive and reliable detection of ORFV DNA. Because the detection limit of our assay was significantly lower than that of conventional PCR, real-time PCR should be especially preferred when detecting pathogens at low copy numbers. Although the variation in the B2L gene sequences of different viral species reaches 15.6% (Gallina et al. 2006; Liu et al. 2013), specificity can be further improved through careful selection of primers. In our study, neither PCR amplification products nor fluorescence signals were observed in the control condition, indicating high specificity of the established real-time PCR assay. Traditional PCR typically shows good performance for specific detection. However, false negatives will occur when the virus content is low. When the virus content is relatively low, the performance of real-time PCR is even better than that of traditional PCR.

SYBR Green I real-time PCR relies on detection of a fluorescent signal using the fluorescent chimeric dye SYBR Green I, eliminating the need for a specific probe. Compared to the TaqMan probe method, it therefore does not require the design of a separate probe, which can be complex and expensive. Moreover, the sensitivity of the SYBR Green I assay compares quite well with the published sensitivity of 50 copies for the TaqMan probe-based real-time PCR assay (Gallina et al. 2006). Indeed, the sensitivity of the SYBR Green I-based assay is even higher than that of the TaqMan probe-based assay, with a detection limit of 20 copies. In addition, the SYBR Green I method provides information regarding the amplification of the PCR reaction in the form of the melting curve. Using the melting curve and Tm value, we could intuitively assess whether the product of the reaction is the intended target. Lastly, the SYBR Green I method also eliminates problems related to probe contamination, in which substandard quality or weak fluorescence signals cause false positive results, or mismatch of the probe with the template, where the lack of a fluorescent signal or low detection rate leads to false negative results.

Thus, our SYBR Green I real-time PCR assay exhibits high specificity for detecting ORFV; among common DNA pathogens, only ORFV returned a positive result. This novel method can detect as few as 20 copies of viral DNA, and the sensitivity is 1000 times higher than that of conventional PCR, effectively protecting against false negative results. In the reproducibility analysis, the inter- and intra-assay CV values ranged from 0.11 to 0.39%, demonstrating that this method possesses high stability and repeatability. Clinical sample testing also showed that real-time PCR exhibits higher sensitivity that conventional PCR in ORFV detection.

In conclusion, our results demonstrate that the established SYBR Green I real-time PCR assay is suitable for diagnosis of ORFV infection in high-throughput clinical samples, epidemiological surveillance, and laboratory research.

## Abbreviations

BHK-21 cell: baby hamster kidney
CPEs: cytopathic effects
DMEM: Dulbecco’s modified Eagle medium
ELISA: enzyme-linked immunosorbent assay
FBS: fetal bovine serum
OFTu: ovine fetal turbinate
ORFV: orf virus
